# Thyroiditis and human blood metabolites: A mendelian randomization study

**DOI:** 10.5937/jomb0-56217

**Published:** 2025-07-04

**Authors:** Lijie Shao, Siqi Liu, Yongfu Song, Shaoyu Han, Yue Ma, Yang Kunpeng, Jingbin Zhang, Bingxue Qi, Yan Guo, Xiaodan Lu

**Affiliations:** 1 Changchun University of Chinese Medicine, College of Integrated Traditional Chinese and Western Medicine, Changchun, China; 2 Precision Medicine Center, Jilin Province People's Hospital, Changchun, China; 3 Shanghai Lixin University of Accounting and Finance, Shanghai, China

**Keywords:** autoimmune thyroiditis, subacute thyroiditis, mendelian randomization analysis, metabolites, autoimuni tiroiditis, subakutni tiroiditis, mendelska analiza randomizacije, metaboliti

## Abstract

**Background:**

The risk factors for thyroiditis, an inflammatory disease with a complex etiology, remain poorly understood. Blood metabolites are known to change during thyroiditis development, suggesting a close relationship between blood metabolites and thyroiditis progression. However, evidence for a causal link is lacking. We employed Mendelian randomization (MR) methodology to systematically investigate the putative causal relationships between blood metabolite profiles and two clinically distinct thyroiditis phenotypes-subacute and autoimmune thyroiditis-providing insights into their metabolic underpinnings.

**Methods:**

We analyzed genomic and health data from 88 million Finnish Biobank participants in the Genome-Wide Association Study (GWAS). The primary analytical method was random-effects inverse variance weighting (IVW), supplemented by the weighted median method (WME) and Mr-Egger. We implemented comprehensive sensitivity analyses encompassing Cochran's Q test, Mr-Egger intercept, leave-one-out analysis (LOO), and Mr-PRESSO to assess heterogeneity, pleiotropy, and outliers. Extended genetic investigations incorporated the linkage disequilibrium score regression (LDSC) method, multivariable Mr (MVMR), and metabolic pathway analyses to provide deeper mechanistic insights.

**Results:**

Ten metabolites were significantly associated with autoimmune thyroiditis, and fifteen with subacute thyroiditis. Nonadecanoate (19:0) and 1-palmitoylglycerophosphoinositol* were found to directly affect subacute thyroiditis. MVMR analyses identified pelargonate (9:0), carnitine, and ADpSGEGDFXAEGGGVR* as having an independent and direct effect on autoimmune thyroiditis. Additionally, metabolic pathways such as neomycin, kanamycin, and gentamicin biosynthesis, histidine metabolism, and starch and sucrose metabolism were linked to autoimmune thyroiditis, while phenylalanine, tyrosine, tryptophan biosynthesis, phenylalanine metabolism, and arginine biosynthesis were associated with subacute thyroiditis.

**Conclusions:**

Our findings establish causal relationships between circulating metabolites and thyroiditis, revealing novel mechanistic insights through integrated genomic and metabolomic analyses. These results not only advance our understanding of thyroiditis pathogenesis but also suggest potential biomarkers for disease screening and therapeutic targets for intervention.

## Abstract (sr)

**Uvod:**

Faktori rizika za tiroiditis, zapaljensko oboljenje složene etiologije, i dalje su slabo shvaćeni. Poznato je da se metaboliti u krvi menjaju tokom razvoja tiroiditisa, što ukazuje na blisku vezu između metabolita u krvi i progresije tiroiditisa. Međutim, nedostaju dokazi o uzročno-posledičnoj vezi. Koristili smo metodologiju Mendelove randomizacije (MR) da sistematski istražimo pretpostavljene uzročne veze između profila metabolita u krvi i dva klinički različita fenotipa tiroiditisa - subakutnog i autoimunog tiroiditisa - pružajući uvid u njihove metaboličke osnove.

**Metode:**

Analizirali smo genomske i zdravstvene podatke od 88 miliona učesnika finske Biobanke u studiji Genome-Vide Association (GVAS). Primarna analitička metoda bila je ponderisanje inverzne varijanse slučajnih efekata (IVV), dopunjeno metodom ponderisane medijane (VME) i Mr-Egger. Implementirali smo sveobuhvatne analize osetljivosti koje obuhvataju Cochran-ov K test, Mr-Egger presretanje, analizu izostavljanja (LOO) i Mr-PRESSO da bismo procenili heterogenost, pleiotropiju i izuzetke. Proširena genetička istraživanja uključila su metodu regresije rezultata neravnoteže veze (LDSC), multivarijabilnu Mr (MVMR) i analize metaboličkog puta da bi se pružila dublji mehanički uvid.

**Rezultati:**

Deset metabolita je bilo značajno povezano sa autoimunim tiroiditisom, a petnaest sa subakutnim tiroiditisom. Utvrđeno je da nonadekanoat (19:0) i 1-palmitoilglicerofosfoinozitol* direktno utiču na subakutni tiroiditis. MVMR analize su identifikovale pelargonat (9:0), karnitin i ADpSGEGDFXAEGGGVR* kao nezavisan i direktan efekat na autoimuni tiroiditis. Pored toga, metabolički putevi kao što su biosinteza neomicina, kanamicina i gentamicina, metabolizam histidina, i metabolizam skroba i saharoze bili su povezani sa autoimunim tiroiditisom, dok su fenilalanin, tirozin, biosinteza triptofana, fenilalanitis povezan sa biosintezom argibaktinom i biosintezom fenilalanina.

**Zaključak:**

Naši nalazi uspostavljaju uzročne veze između cirkulirajućih metabolita i tiroiditisa, otkrivajući nove mehaničke uvide kroz integrisane genomske i metabolomske analize. Ovi rezultati ne samo da unapređuju naše razumevanje patogeneze tiroiditisa, već takođe sugerišu potencijalne biomarkere za skrining bolesti i terapijske ciljeve za intervenciju.

## Introduction

Thyroiditis encompasses a spectrum of diseases characterized by inflammation of the thyroid gland, with autoimmune thyroiditis (AIT) being the most prevalent form, exemplified by Hashimoto’s thyroiditis (HT). AIT includes various clinical-pathological entities beyond the classic form, such as juvenile thyroiditis, Riedel’s thyroiditis, IgG4 thyroiditis, painless thyroiditis, postpartum thyroiditis, Hashimoto’s encephalopathy, and fibrous variants characterized by glandular fibrosis and rapid progression toward hypothyroidism [1]. Subacute thyroiditis, typically following an upper respiratory viral infection, is marked by transient hyperthyroidism with anterior neck pain, suppressed thyroid-stimulating hormone (TSH) levels, and low radioiodine uptake, usually resolving spontaneously within a few months [2]. Thyroiditis is more common in women, increases with age, and is often associated with other autoimmune diseases [3].

Blood metabolites have been established as fundamental biomarkers in clinical practice, specifically in thyroid disease management. Thyroid dysfunction has been shown to significantly affect metabolic homeostasis, whereby distinct patterns of metabolic alterations serve as potential diagnostic and prognostic indicators. Thyroid hormones have been demonstrated to regulate lipid metabolism, as substantiated by the well-documented association between thyroid status and serum lipid profiles [4]. Alterations in thyroid function exhibit distinct effects on lipid homeostasis: hypothyroid states are characterized by elevated serum concentrations of total cholesterol, LDL-C, and triglycerides, whereas hyperthyroidism induces reciprocal reductions in these circulating lipid parameters [5]. These metabolic disruptions have been found to not only reflect thyroid dysfunction but also to contribute to disease progression through complex feedback mechanisms that regulate fatty acid and cholesterol metabolism [3] [4].

Mendelian randomization (MR) employs genetic variants as instrumental variables to establish causal inference between putative exposure factors and disease outcomes, thereby minimizing confounding effects inherent in observational studies. Based on the principle of »natural random assignment« in genetics, MR leverages the random inheritance of genetic variants from parents to offspring, analogous to group randomization in controlled trials [6]. This methodological framework has fundamentally transformed our understanding of disease etiology by establishing robust evidence for causal relationships that were previously limited to correlational observations. The strategic utilization of exposure-associated genetic variants as instrumental variables in Mendelian randomization enables robust causal inference by circumventing both confounding factors and reverse causality that typically confound observational studies [7] [8]. Compared to traditional observational studies, MR offers significant advantages: it addresses confounding factors unaffected by social, environmental, or behavioral influences; it circumvents reverse causation due to the fixed nature of genetic variants from birth; and it is both time-efficient and cost-effective when randomized controlled trials are impractical [9]. This approach has demonstrated significant utility in identifying novel therapeutic targets and validating existing ones, subsequently expediting the drug development process and enhancing the efficacy of public health interventions.

Epidemiological investigations have revealed robust correlations between specific circulating metabolites and thyroid pathologies, with particular emphasis on Hashimoto’s thyroiditis, where distinct metabolic signatures intersect with immunological markers and thyroid autoantibody profiles [10] [11]. In this study, we leveraged Mendelian randomization methodology to interrogate the causal associations between thyroiditis and specific blood metabolites. This systematic approach unveils previously unrecognized pathogenic mechanisms and presents promising avenues for both diagnostic advancement and therapeutic development

## Materials and methods

### Research design

**Figure 1:**
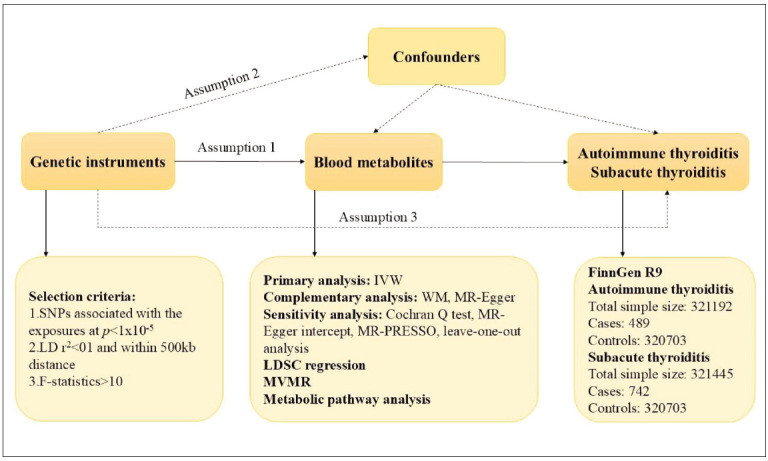
The detailed description of the study design.

This study utilized ethically approved, publicly available datasets accessed through established database repositories, adhering to standardized data protection protocols and institutional review requirements. Valid causal inference through Mendelian randomization requires satisfaction of three critical assumptions: first, genetic instruments must demonstrate strong association with the exposure (relevance); second, instruments must be independent of confounding variables (independence); and third, genetic variants must influence outcomes exclusively through the exposure pathway (exclusion restriction) [12]. We conducted comprehensive Mendelian randomization analyses utilizing genome-wide association studies (GWAS) from Finnish cohorts, systematically evaluating the causal influence of 486 circulating metabolites (exposures) on thyroiditis risk (outcome) [13]. The study workflow is depicted in Figure 1.

### Blood metabolite GWAS resources

We obtained genetic association data for blood metabolites from the Metabolomics GWAS Server (http://metabolomics.helmholtz-muenchen.de/gwas/). This resource incorporates the landmark study by Shin et al. [14], which remains one of the most extensive analyses of genetic determinants governing human blood metabolites. By integrating genome-wide association studies (GWAS) withmetabolomics, the research elucidated complex relationships between metabolic pathways and genetic variation. Utilizing samples from two European cohorts—the Twins UK study in the United Kingdom and the KORA cohort in Germany—comprising a total of 7,824 adults, the study integrated gene expression data, metabolite heritability, and drug target associations. A network model was constructed to represent interactions between metabolites and genes, ultimately identifying approximately 2.1 million single nucleotide polymorphisms (SNPs) associated with 486 metabolites and human genetic variation [14]. Of these metabolites, 309 with known chemicalproperties were categorized into eight broad classes—amino acids, carbohydrates, cofactors and vitamins, energy, lipids, nucleotides, peptides, and xenobiotics—as recorded in the Kyoto Encyclopedia of Genes and Genomes (KEGG) database [15].

### GWAS data on thyroiditis

GWAS data for thyroiditis were obtained from the Finnish Biobank (https://r9.risteys.finngen.fi). Two phenotypes of interest, autoimmune thyroiditis and subacute thyroiditis, were selected for analysis. The autoimmune thyroiditis cohort consisted of 489 cases and 320,703 controls, while the subacute thyroiditis cohort had 742 cases, with the same control group (Table 1).

**Table 1:** Characteristics of the Summary Datasets for thyroiditis.

NO. Phenotype	Ethnic	SNPs	case	control	Sample size	Prevalence (%)
Autoimmune thyroiditis	Finland	20168706	489	320703	321192	0.15
Subacute thyroiditis	Finland	20168773	742	320703	321445	0.23

### Instrumental variable identification

Multiple stringent criteria were applied to identify robust genetic instruments for blood metabolite levels. The metabolite selection process was conducted based on three key criteria: (1) SNP-metabolite associations that achieved genome-wide significance (P < 1 × 10^-5^), (2) genetic instrument strength as indicated by F-statistics > 10, and (3) genetic instrument independence as determined by LD R^2^ < 0.1, with a minimum requirement of three independent SNPs per metabolite. To establish significant associations between SNPs and metabolite biomarkers, we implemented a stringent significance threshold (P < 1 × 10^-5^). The stringent threshold guaranteed that only strongly associated SNPs served as genetic instruments, strengthening the reliability of downstream Mendelian randomization inference. We then applied a strict linkage disequilibrium (LD) criterion, with an R^2^ value below 0.1 within a 500 KB window. To ensure independent genetic effects, we eliminated potential SNP-SNP interactions, thereby maintaining the validity of each instrumental variable. By applying these parameters, we optimized the reliability and precision of the analysis, ultimately improving the validity of the results obtained. F-statistics were calculated to assess instrument strength and mitigate weak instrument bias. The F-statistic decreases as the number of SNPs increases but increases with larger sample sizes and greater SNP explanatory power over the exposure. Metabolites yielding F-statistics below 10 were excluded, and SNPs showing strong associations with outcomes (P < 1 × 10^-5^) were removed to prevent genetic confounding. To ensure robust causal estimation and minimize confounding, we performed MR analyses exclusively on metabolites with at least three independent genetic instruments.

### Analytical methods and sensitivity analysis

When the three core assumptions of the instrumental variable (IV) method are satisfied, the inversevariance weighting (IVW) approach provides precise causal effect estimates and is considered reliable for causal inference [16]. The IVW method served as our primary analytical approach for evaluating metabolite-thyroiditis causal relationships, adopting a conventional significance threshold (P < 0.05). Findings were validated using complementary MR methods, including MR-Egger regression and WME. These complementary approaches offer distinct methodological advantages: WME remains reliable when up to half of the genetic instruments are invalid [17], while MR-Egger addresses pleiotropy bias in genetic variants, offering robust causal effect estimates [18]. The convergence of findings across three distinct MR approaches (IVW, MR-Egger, and WME) provides robust support for the identified causal relationships.

Robustness was evaluated through multiple sensitivity analyses, encompassing Cochran’s Q test, MR-Egger intercept, LOO, and MR-PRESSO. These complementary approaches rigorously evaluated potential confounders, including outlier effects, statistical heterogeneity, and directional pleiotropy. Heterogeneity was determined significant at P < 0.05 using Cochran’s Q statistic [19]. The MR-Egger intercept tested for directional pleiotropy; a significant deviation of the intercept from zero indicated its presence [20]. LOO analysis evaluated the impact of individual genetic variants on causal estimates, increasing the credibility of the analysis [20]. MR-PRESSO identified horizontal pleiotropy and enhanced result robustness by detecting and correcting for pleiotropic outliers [21].

A comprehensive set of screening criteria was developed to ensure methodological rigor and transparency in metabolite selection, incorporating both statistical significance and validation parameters: (1) Primary statistical threshold: statistically significant causal effects as determined by IVW analysis (P < 0.05); (2) Methodological consistency: concordant effect estimates obtained across IVW, MR-Egger, and WME approaches; (3) Robustness validation: demonstrated by the absence of heterogeneity in Cochran’s Q test (P > 0.05), the lack of directional pleiotropy in MR-Egger intercept test, and the absence of outliers in MR-PRESSO analysis; (4) Stability confirmation: verified through consistent estimates in leave-one-out sensitivity analyses. Statistical power was evaluated using an online tool (https://shiny.cnsgenomics.com/mRnd/), validating the robustness of causal estimates [20]. This tool computes power to detect causal effects from instrumental variables based on asymptotic theory and known study parameters. Power calculations incorporated instrumental variable R^2^, case proportions, and IVW-derived odds ratios (ORs), with a set at 0.05.

### Directionality and genetic relationship analysis

Although SNPs associated with thyroiditis were excluded when selecting IVs, factors like population stratification and cryptic relatedness could still cause false positives, contributing to thyroiditis genetic risk. Genetic correlations between metabolites and thyroiditis were quantified using LDSC. LDSC estimates the SNP heritability of complex traits or diseases by regressing the GWAS summary statistics (e.g., Zscores or chi-square values) on LD scores. This approach identifies the polygenicity of genetic variants and reduces systematic bias. SNPs with high LD scores capture more genetic variation, making them more likely to be associated with traits. Using this method, we evaluated SNP heritability across the genome and assessed genetic correlations between traits, correcting for biases from population stratification and other factors [22].

Bidirectional causality was investigated through reverse MR analyses of metabolites showing primary causal associations with thyroiditis. Reverse MR analyses employed identical methodological parameters as the primary analysis.

### Multivariate MR analysis

The validity of Mendelian randomization inference relies critically on genetic variants exhibiting exclusive associations with the exposure of interest. However, single nucleotide polymorphisms (SNPs) may influence outcomes through multiple pathways—a phenomenon known as horizontal pleiotropy—which violates instrumental variable (IV) assumptions and leads to biased causal estimates. To disentangle direct causal relationships, we employed MVMR approach, enabling simultaneous assessment of multiple exposures while adjusting for shared genetic architecture. This approach allowed us to account for and adjust complex interactions, enhancing the reliability of our MR analysis [23].

The MVMR approach utilized two methods: inverse variance weighting (IVW) [24] and MR-PRESSO [21]. In MVMR, IVW combines the weighted effects of multiple genetic variants, improving statistical efficiency and minimizing random error, thereby enhancing the reliability of causal inference and mitigating pleiotropy interference. To mitigate bias from horizontal pleiotropy, we implemented MR-PRESSO for systematic detection and removal of genetic instrument outliers.

### Pathway-based metabolomic analysis

To interpret the biological architecture underlying metabolite-thyroiditis associations, we implemented systematic pathway-based functional analysis. KEGG-annotated metabolites underwent systematic pathway enrichment analysis via the MetaboAnalyst platform (https://www.metaboanalyst.ca/). Pathway analysis elucidated the molecular mechanisms underlying the identified metabolite-disease associations.

## Results

### Preliminary and sensitivity analyses

**Figure 2:**
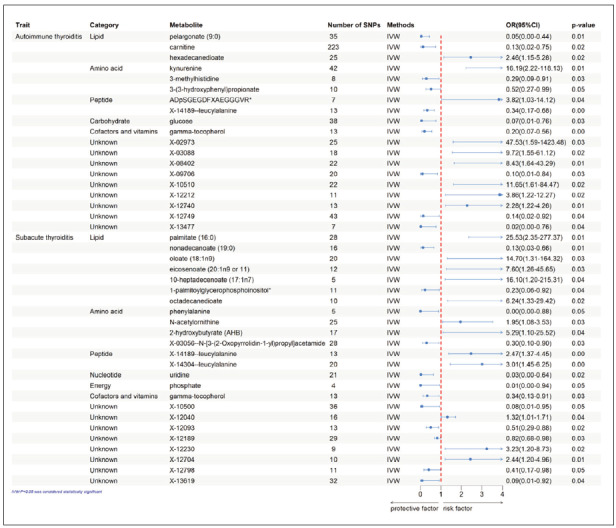
Forest plot for the causality of blood metabolites on autoimmune thyroiditis and subacute thyroiditis derived from inverse variance weighted (IVW) analysis. CI, confidence interval; OR, odds ratio; SNPs, single nucleotide polymorphisms.

Mendelian randomization analysis was conducted across 486 serum metabolites using stringentinstrumental variable criteria. All metabolite-related SNPs had F-statistic values above 10, confirming strong genetic proxies for causal inference. Eighteen metabolites were preliminarily identified as potentially causally related to autoimmune thyroiditis through IVW analysis. After complementary and sensitivity analyses, as well as power calculations, stringent filtering identified 10 metabolites as putative causal candidates (Table 2), including pelargonate (9:0) (OR 0.05, 95% CI: 0.00-0.44, P=0.008), kynurenine (OR 16.19, 95% CI: 2.22-118.13, P=0.006), carnitine (OR 0.13, 95% CI: 0.02-0.75, P=0.022), 3-methylhistidine (OR 0.29, 95% CI:0.09-0.91 ,P=0.034), glucose (OR 0.07, 95% CI: 0.01-0.76, P=0.028), gamma-tocopherol (OR 0.20, 95% CI: 0.07-0.56, P=0.002), ADpSGEGDFXAEGGGVR* (OR 3.82, 95% CI: 1.03-14.12, P=0.045), 3-(3-hydroxyphenyl)propionate (OR 0.52, 95% CI: 0.27–0.99, P=0.048), hexadecanedioate (OR 2.46, 95% CI: 1.15–5.28, P=0.020), leucylalanine (OR 0.34, 95% CI: 0.17–0.68, P=0.002). IVW analysis initially yielded 21 metabolites associated with subacute thyroiditis, with 11 characterized metabolites remaining significantafter rigorous validation, including phenylalanine(OR 0.00, 95% CI: 0.00–0.88, P=0.046), nonadecanoate (19:0) (OR 0.13, 95% CI: 0.03–0.66, P=0.014), oleate (18:1n9) (OR 14.70, 95% CI: 1.31–164.32, P=0.029), phosphate (OR 0.01, 95% CI: 0.00–0.94, P=0.047), N-acetylornithine (OR 1.95, 95% CI: 1.08–3.53, P=0.027), gamma-tocopherol (OR 0.34, 95% CI: 0.13–0.91, P=0.032), eicosenoate (20:1n9 or 11) (OR 7.60, 95% CI: 1.26-45.65, P=0.027), 10-heptadecenoate (17:1n7) (OR 16.10, 95% CI: 1.20–2.153100e+02, P=0.036), 1-palmitoylglycerophosphoinositol*( OR 0.23, 95% CI:0.06–0.92, P=0.038), leucylalanine(OR 2.47, 95% CI:1.37–4.45,P=0.003), leucylalanine (OR 3.01, 95% CI:1.45–6.25, P=0.003) (Figure 2). Causal estimates remained robust across multiple MR methods, with IVW analyses yielding significant associations (P < 0.05) that were directionallyconsistent with MR-Egger and WME. Following outlier removal, the MR-PRESSO analysis revealed that none of the genetic variants exhibited horizontal pleiotropy. No evidence of heterogeneity or pleiotropy was detected by Cochran’s Q test and MR-Egger intercept analysis (both P > 0.05). Additionally, we calculated the estimated statistical power and the results demonstrated that all estimated statistical powers exceeded 0.8, indicating adequacy. This methodological consistency reinforces the robustness of our observed associations and supports the reliability of our conclusions. Our Mendelian randomization analyses establish previously unidentified causal relationships between circulating metabolites and thyroid inflammation. Sequential omission of individual SNPs through LOO confirmed the stability of causal estimates, indicating absence of influential outlier variants. The convergence of multiple analytical approaches substantiates the causal relationship between targeted blood metabolites and thyroiditis pathogenesis.

**Table 2:** Supplementary and sensitivity analyses for causality from blood metabolites on thyroiditis.

Trait	Metabolites		MR analysis			Heterogeneity		Pleiotropy		power
			Methods	OR (95%CI)	P	Q	P	Intercept	p	
Autoimmune<br>thyroiditis	Lipid									
		pelargonate (9:0)	ME	0.01<br>(0.00–8.00)	.18	32.81	.48	0.02	.58	1.00
			WM	0.01<br>(0.00–0.33)	.01					
		carnitine	ME	0.01<br>(0.00–0.87)	.04	224.29	.43	0.01	.23	1.00
			WM	0.19<br>(0.01–3.46)	.26					
		hexadecanedioate	ME	2.84<br>(0.88–9.17)	.09	23.26	.45	-0.01	.76	1.00
			WM	3.56<br>(1.18–10.77)	.02					
	Amino acid									
		kynurenine	ME	4.58<br>(0.07–299.41)	.48	47.15	.20	0.02	.50	1.00
			WM	2.40<br>(0.14–42.13)	.55					
		3-methylhistidine	ME	0.38<br>(0.01–10.85)	.59	9.67	.14	-0.02	.86	0.97
			WM	0.47<br>(0.12–1.83)	.28					
		3-(3-hydroxyphenyl)<br>propionate	ME	0.84<br>(0.14–4.98)	.85	9.60	.29	-0.04	.59	1.00
			WM	0.57<br>(0.23–1.38)	.21					
	Peptide									
		ADpSGEGDFXAEGGGVR*	ME	4.37<br>(0.10–196.91)	.48	1.39	.92	-0.01	.94	1.00
			WM	3.78<br>(0.78–18.25)	.10					
		X-14189--leucylalanine	ME	0.43<br>(0.12–1.53)	.22	7.92	.72	-0.02	.65	1.00
			WM	0.35<br>(0.12–1.01)	.05					
	Carbohydrate									
		glucose	ME	0.04<br>(0.00–3.38)	.17	29.86	.75	0.01	.77	1.00
			WM	0.09<br>(0.00–3.14)	.18					
	Cofactors and<br>vitamins									
		gamma-tocopherol	ME	0.27	.27	7.76	.73	-0.01	.76	1.00
			WM	0.19<br>(0.05–0.76)	.02					
Subacute<br>thyroiditis	Lipid									
		nonadecanoate<br>(19:0)	ME	0.16<br>(0.01–3.45)	.26	13.59	.48	-0.01	.87	1.00
			WM	0.31<br>(0.03–3.37)	.33					
		oleate (18:1n9)	ME	1313.26<br>(0.17–9943323.52)	.13	21.26	.27	-0.07	.32	1.00
			WM	16.43<br>(0.59–459.90)	.10					
		eicosenoate<br>(20:1n9 or 11)	ME	1.20<br>(0.01–147.85)	.94	13.45	.20	0.05	.44	1.00
			WM	7.97<br>(0.84–75.78)	.07					
		10-heptadecenoate<br>(17:1n7)	ME	5786.26<br>(0.00–5.571196e+10)	.37	2.96	.40	-0.12	.52	1.00
			WM	9.23<br>(0.32–2.658600e+02)	.19					
		1-palmitoylglycerophosphoinositol*	ME	0.45<br>(0.02–12.61)	.65	3.89	.92	-0.02	.69	1.00
			WM	0.22<br>(0.04–1.31)	.10					
		octadecanedioate	ME	2.38<br>(0.10–56.05)	.61	2.14	.98	0.03	.51	1.00
			WM	3.42<br>(0.50–23.30)	.21					
	Amino acid									
		phenylalanine	ME	0.12<br>(0.00–1776049.07)	.82	1.23	.75	-0.05	.53	1.00
			WM	0.00<br>(0.00–0.82)	.05					
		N-acetylornithine	ME	1.72(0.71–4.19)	.24	28.95	.18	0.01	.71	1.00
			WM	1.86<br>(0.90–3.83)	.09					
		2-hydroxybutyrate<br>(AHB)	ME	19.49<br>(0.51–746.32)	.13	14.89	.46	-0.03	.45	1.00
			WM	4.05<br>(0.39–42.43)	.24					
		X-03056-N-[3-(2-Oxopyrrolidin-1-yl)propyl]acetamide	ME	0.24<br>(0.03–1.86)	.18	19.94	.79	0.01	.80	1.00
			WM	0.38<br>(0.08–1.72)	.21					
	Peptide									
		X-14189-leucylalanine	ME	1.09<br>(0.39–3.06)	.87	9.30	.59	0.06	.09	1.00
			WM	1.63<br>(0.72–3.68)	.24					
		X-14304--eucylalanine	ME	2.16<br>(0.22–21.06)	.52	19.04	.39	0.01	.76	1.00
			WM	2.75<br>(0.96–7.87)	.06					
	Nucleotide									
		uridine	ME	0.01<br>(0.00–78.21)	.33	18.96	.46	0.01	.79	1.00
			WM	0.01<br>(0.00–0.87)	.04					
	Energy									
		phosphate	ME	0.05<br>(0.00–417.55)	.58	2.00	.37	-0.02	.74	1.00
			WM	0.05<br>(0.00–16.55)	.31					
	Cofactors and<br>vitamins									
		gamma-tocopherol	ME	0.35<br>(0.04–2.99)	.36	15.78	.15	0.00	.99	1.00
			WM	0.27<br>(0.08–0.90)	.03					

### Directionality and genetic relationship analysis

Using the LDSC approach, we found strong genetic correlations between autoimmune thyroiditis and two specific blood metabolites: kynurenine (Rg=0.577, Se=0.170, P=0.001) and 3-methylhistidine (Rg=-1.730, Se=0.840, P=0.039). We also found strong genetic correlation between subacute thyroiditis and the blood metabolite octadecanedioic (Rg=-0.780, Se=0.341, P=0.022). Analysis of metabolite-genetic architecture uncovered mechanistic links that may inform both preventive approaches and therapeutic strategies for thyroiditis.

### MVMR

**Figure 3:**
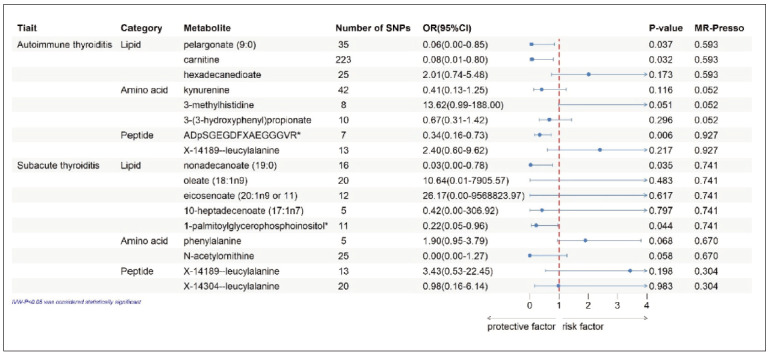
Multivariable Mendelian randomization analysis of the final identified blood metabolites. 95%CI, 95% confidence interval; OR, odds ratio; MVMR, Multivariable Mendelian randomization; MR-PRESSO, MR-Pleiotropy RESidual Sum and Outier; SNPs, single nucleotide polymorphisms.

To address metabolite intercorrelations, we employed multivariable Mendelian randomization,enabling assessment of exposure-specific causal effects. MVMR analysis resolved the independent contributions of metabolic exposures, distinguishing direct from indirect effects on thyroiditis risk. MVMR estimates obtained by the IVW method indicated that the genetically predicted levels of pelargonate (9:0), carnitine and ADpSGEGDFXAEGGGVR* independently affected autoimmune thyroiditis. In addition, nonadecanoate (19:0) and 1-palmitoylglycerophosphoinositol* were found to independently affect subacute thyroiditis (Figure 3).

### Metabolic pathway analysis

In autoimmune thyroiditis, our findings highlight a key role for glucose in the biosynthetic metabolic pathways of neomycin, kanamycin and gentamicin biosynthesis and in the starch and sucrose metabolism. Within the histidine metabolic network, 3-methylhistidine serves as a crucial regulatory metabolite. Similarly, in subacute thyroiditis, we revealed a key role for phenylalanine in the biosynthetic metabolic pathways of Phenylalanine, tyrosine and tryptophan biosynthesis and in the Phenylalanine metabolism, in addition to N-acetylornithine in the Arginine biosynthesis (Table 3). These profound associations were statistically significant (P<0.05). This suggests that comprehensive understanding these metabolites and their roles in related metabolic processes may help uncover the underlying mechanisms of these diseases and inform new therapeutic strategies.

**Table 3:** Metabolic pathways with significant enrichment of blood metabolites.

Item	Metabolic pathways	Involved metabolites	p value
Autoimmune thyroiditis	Neomycin, kanamycin and gentamicin biosynthesis	D-Glucose	0.0050745
	Histidine metabolism	N(pi)-Methyl-L-histidine	0.040057
	Starch and sucrose metabolism	D-Glucose	0.044979
Subacute thyroiditis	Phenylalanine, tyrosine and tryptophan biosynthesis	L-Phenylalanine	0.0076045
	Phenylalanine metabolism	L-Phenylalanine	0.01517
	Arginine biosynthesis	N-Acetylornithine	0.026447

## Discussion

We performed systematic Mendelian randomization analysis across 486 blood metabolites to identify their causal relationships with thyroiditis, focusing on autoimmune and subacute thyroiditis. We leveraged genetic variants as IVs, combining metabolomic GWAS data (http://metabolomics.helmholtzmuenchen.de/gwas/) with thyroiditis statistics from FinnGen R9 through IVW analysis. Our preliminary study identified correlations between 18 metabolites and autoimmune thyroiditis and 21 metabolites associated with subacute thyroiditis. We assessed the heterogeneity and sensitivity of these metabolites. High genetically determined levels of pelargonate (9:0), carnitine, 3-methylhistidine, glucose, gamma-tocopherol, 3-(3-hydroxyphenyl)propionate, and leucylalanine were associated with a reduced risk of autoimmune thyroiditis, whereas elevated levels of kynurenine, ADpSGEGDFXAEGGGVR*, and hexadecanedioate were linked to an increased risk. Similarly, higher concentrations of nonadecanoate (19:0), 1-palmitoylglycerophosphoinositol*, phenylalanine, phosphate, and gamma-tocopherol were associated with a decreased risk of subacute thyroiditis, while elevated levels of oleate (18:1n9), N-acetylornithine, eicosenoate (20:1n9 or 11), 10-heptadecenoate (17:1n7), and leucylalanine were linked to an increased risk. These findings suggest that elucidating the metabolic pathways regulating these biomolecules could enhance our understanding of thyroiditis pathogenesis and aid in identifying new diagnostic and therapeutic targets [24].

Thyroid hormones (THs), particularly thyroxine (T4) and triiodothyronine (T3), are master regulatorsof regulating metabolic processes such as lipid synthesis, catabolism, and mobilization [25] [26]. In a healthy state, the thyroid gland maintains lipid metabolism balance by promoting cholesterol transport and fatty acid oxidation [27]. Normal thyroid function is essential for sustaining appropriate lipid levels. However, thyroid dysfunction leads to prominent lipid metabolism abnormalities. Hypothyroidism is often associated with elevated lipid levels, evidenced by increased plasma cholesterol, triacylglycerol, and lowdensity lipoprotein cholesterol (LDL-C) [28]. In patients with subclinical hypothyroidism, lipid metabolism deterioration is more pronounced, particularly with elevated thyroid-stimulating hormone (TSH), potentially heightening the risk of cardiovascular disease [28] [29]. TSH levels significantly correlate with elevated total cholesterol (TC) and LDL-C, likely due to regulation of hepatic lipoprotein metabolism [30]. Hypothyroidism is also linked to a reduction in highdensity lipoprotein (HDL), exacerbating cardiovascular risk [28] [31]. While lipid reductions are commonly observed in hyperthyroid patients, excessive mobilization of lipid metabolism can result in metabolic disorders [32] [33]. THs regulate lipid metabolism through multiple molecular pathways, with the thyroid-hepatic axis playing a key role [31]. They influence lipid metabolism via direct gene regulation and cross-signaling with nuclear receptors such as peroxisome proliferator-activated receptor (PPAR) and liver X receptor (LXR). The effect of THs on insulin sensitivity is also crucial in lipid metabolism regulation [27]. Subclinical thyroid dysfunction does not always improve lipid metabolism, even with levothyroxine treatment [28] [29], highlighting the need for novel treatment strategies, particularly in early stages of thyroid dysfunction.

Carnitine (L-carnitine), a natural water-soluble quaternary ammonium compound widely distributed in mammalian tissues and mainly stored in skeletal muscles, accounts for 95% of total body carnitine. It is crucial in the β-oxidation of long-chain fatty acids, enabling their entry into the mitochondrial matrix for metabolism [34] [35]. THs regulate energy metabolism, establishing a close relationship between carnitine levels and thyroid function. Our study suggests that carnitine may directly influence autoimmune thyroiditis progression and act as a protective factor against inflammation in autoimmune thyroid disease. THs promote long-chain fatty acid oxidation by regulating carnitine palmitoyltransferase I (CPT I) expression [36]. In hyperthyroidism, γ-butyrobetaine hydroxylase (BBH) activity increases, promoting carnitine biosynthesis; in hypothyroidism, BBH activity decreases [37]. This supports the idea that THs regulate carnitineavailability by influencing key steps in fat metabolism. Carnitine acts as a peripheral TH antagonist, preventing T3 and T4 from entering the nucleus and inhibiting their effects [34] [38]. Cell experiments show that carnitine significantly inhibits TH nuclear uptake, particularly in neurons and liver cells, blocking TH signaling at the cellular level [38]. Thyroid dysfunction often results in abnormal carnitine levels, contributing to muscle weakness. Patients with hypothyroidism and hyperthyroidism have lower skeletal muscle carnitine levels, which increase when thyroid function normalizes [39]. Additionally, L-carnitine supplementation can effectively reduce fatigue symptoms, especially mental fatigue, in hypothyroid patients receiving levothyroxine treatment [40], highlighting its supplementary role in thyroid disease treatment.

The fibrinogen-cleaving peptide ADpSGEGDFXAEGGGVR* emerges as a putative risk factor forautoimmune thyroiditis, although supporting studies are limited. TH secretion and regulation involve a complex network of polypeptides and their receptors. These peptides mediate critical thyroid signaling cascades and disease mechanisms, including autoimmune thyroid diseases and other metabolic disorders [41] [42]. TSH is a crucial peptide hormone regulating thyroid function by binding to the TSH receptor (TSHR) in thyroid cells, influencing T4 and T3 synthesis and secretion [43]. The interaction of TSH with its receptor activates adenylyl cyclase through G proteincoupled receptors and regulates thyroid cell proliferation and function [44]. Non-traditional peptides, suchas ghrelin, affect thyroid function through TSHR by inhibiting TSH-induced thyroglobulin and thyroid peroxidase (TPO) mRNA expression, thus inhibiting TH production [41]. In autoimmune thyroid diseases like Graves’ disease and Hashimoto’s thyroiditis, anti-TSHR antibodies (TSHR-Ab) are core pathological factors, modulating TSHR activity and leading to hyperthyroidism or hypothyroidism. Other blocking or neutralizing antibodies contribute to hypothyroidism by inhibiting TSH effects [42]. Certain peptides play significant roles in regulating autoimmune responses; for example, the human thyroglobulin (hTg) p2340 peptide can induce autoimmune thyroiditis in HLADR3 transgenic mice [45], indicating that some thyroid- related peptides can induce T cell-mediated immune responses via HLA-DR binding, leading to thyroid tissue damage [45] [46]. Increased expression of chemokines like CCL2 and CCL3 in Hashimoto’s thyroiditis patients suggests their role in inflammatory responses and tissue damage by attracting immune cells to the thyroid gland [47]. Peptides play a pivotal role in regulating physiological functions and pathological development of the thyroid gland, especially in autoimmune thyroid diseases. Thorough investigation of these peptides and their receptors is expected to lead to more precise targeted therapies for efficient thyroid disorder management and treatment [46] [48].

Our study suggests that pelargonate may act as a protective factor in autoimmune thyroiditis, directly influencing disease progression. However, this finding lacks confirmation from other studies, making it a potentially significant discovery offering new research perspectives. Similarly, nonadecanoate and 1-palmitoylglycerophosphoinositol* may directly influence subacute thyroiditis development, acting independently of other factors. Since no relevant studies have validated these findings, they represent significant new discoveries warranting further investigation.

This study offers several advantages. By integrating metabolomics and genomics, we employedMendelian randomization to delineate causal relationships between serum metabolites and autoimmune and subacute thyroiditis. The study utilized large sample sizes and single nucleotide polymorphisms (SNPs) from reputable consortia, ensuring data quality. The techniques used to evaluate causal relationships—IVW, weighted median, and MR-Egger—are well-established and reliable, providing extensive data for further research.

Several methodological limitations were identified in this investigation. Although the Mendelian randomization approach reduces confounding factors, residual pleiotropy remains a concern, given that genetic variants could influence thyroiditis through pathways that operate independently of metabolite levels. The employed genetic instruments potentially provide incomplete representation of metabolite-disease relationships, specifically in cases where metabolites exhibit weak genetic associations or are strongly influenced by environmental factors. The generalizability of findings is constrained by the predominant utilization of European ancestry data, which may fail to capture population-specific genetic variations. The cross-sectional nature of the study design precludes temporal analysis of metabolite changes throughout disease progression, whereas limitations in sample size and absence of validation cohorts warrant cautious interpretation of results. Further investigations should emphasize multi-ethnic cohorts, longitudinal metabolomic analyses, gene-environment interactions, and tissue-specific metabolomic profiling to enhance the robustness of the evidence base.

Mendelian randomization analyses revealed distinct metabolic signatures and pathways that demonstrate causal influences on thyroiditis pathogenesis, yielding novel mechanistic insights with implications for current therapeutic strategies. In subacute thyroiditis, we identified causal effects of nonadecanoate (19:0) and 1-palmitoylglycerophosphoinositol*, accompanied by significant alterations in amino acid biosynthesis pathways, particularly those involving phenylalanine, tyrosine, tryptophan, and arginine. These findings suggest potential therapeutic synergies with established anti-inflammatory interventions. For autoimmune thyroiditis, MVMR analyses revealed independent causal roles of pelargonate (9:0), carnitine, and ADpSGEGDFXAEGGGVR*, alongside perturbations in aminoglycoside biosynthesis and histidine metabolism pathways, indicating possible mechanisms to enhance current immunomodulatory approaches.

The identified metabolic signatures may function as complementary diagnostic tools alongside conventional markers, such as thyroid antibodies and inflammatory indicators, facilitating enhanced precision in disease monitoring and treatment response assessment. The observed pathway-specific alterations advance both the mechanistic understanding of thyroiditis pathogenesis and the identification of novel therapeutic targets, with particular relevance for cases refractory to conventional therapeutic approaches. Although these findings present promising avenues for biomarker development and therapeutic innovation, rigorous prospective clinical validation studies remain essential for establishing clinical utility. Future investigations should prioritize the integration of these metabolic insights into established treatment paradigms, with the objective of optimizing therapeutic outcomes in thyroiditis management.

## Dodatak

### Data availability statement

All experimental datasets described herein are stored in public repositories, with additional information available from the authors upon request.

### Author contributions

The study was conceived by LJS and YFS. Data collection was carried out by YFS and YM. LJS and SYH were responsible for data processing and analysis. The manuscript was drafted by LJS, with revisions contributed by SQL. XDL and YG jointly supervised the entire study and contributed to finalizing the manuscript. All authors have read and approved the final manuscript.

Lijie Shao and Siqi Liu contributed equally to this work. 

### Funding

The role of VEGF A in the pathogenesis and progression of chronic lymphocytic thyroiditis in the thyroid gland of Jilin Province (YDZJ202201 ZYTS 148).

Capital Construction Projects in the Provincial Budget in 2023 »The Development and application of accurate diagnosis for primary hyperparathyroidism of parathyroid gland« (2023C041-1).

2024 Jilin Provincial Science and Technology Department Project: »Establishment of AI PredictionModels for Immune Characteristics of Infectious Diseases and Research on Their Metabolic Mechanisms« (202401007JJ).

### Acknowledgments

We gratefully acknowledge the FinnGen and UK Biobank consortia for generously sharing publicly available GWAS summary-level statistics.

### Conflict of interest statement

All the authors declare that they have no conflict of interest in this work.
